# miRNA-36 inhibits KSHV, EBV, HSV-2 infection of cells via stifling expression of interferon induced transmembrane protein 1 (IFITM1)

**DOI:** 10.1038/s41598-017-18225-w

**Published:** 2017-12-21

**Authors:** Hosni A. M. Hussein, Shaw M. Akula

**Affiliations:** Department of Microbiology & Immunology, Brody School of Medicine at East Carolina University, Greenville, NC 27834 USA

## Abstract

Kaposi’s sarcoma-associated herpesvirus (KSHV) is etiologically associated with all forms of Kaposi’s sarcoma worldwide. Little is currently known about the role of microRNAs (miRNAs) in KSHV entry. We recently demonstrated that KSHV induces a plethora of host cell miRNAs during the early stages of infection. In this study, we show the ability of host cell novel miR-36 to specifically inhibit KSHV-induced expression of interferon induced transmembrane protein 1 (IFITM1) to limit virus infection of cells. Transfecting cells with miR-36 mimic specifically lowered IFITM1 expression and thereby significantly dampening KSHV infection. In contrast, inhibition of miR-36 using miR-36 inhibitor had the direct opposite effect on KSHV infection of cells, allowing enhanced viral infection of cells. The effect of miR-36 on KSHV infection of cells was at a post-binding stage of virus entry. The highlight of this work was in deciphering a common theme in the ability of miR-36 to regulate infection of closely related DNA viruses: KSHV, Epstein-Barr virus (EBV), and herpes simplexvirus-2 (HSV-2). Taken together, we report for the first time the ability of host cell miRNA to regulate internalization of KSHV, EBV, and HSV-2 in hematopoietic and endothelial cells.

## Introduction

Kaposi’s sarcoma-associated herpesvirus (KSHV) causes Kaposi’s sarcoma (KS)^1^. To a lesser extent, KSHV is etiologically associated with rare neoplastic disorders like primary effusion lymphoma (PEL), and multicentric Castleman disease (MCD)^2^. KS is a malignant vascular tumor characterized by lesions occurring mainly on the skin, but can also affect the mucosa and visceral organs^3^. Hallmarks of KS are angiogenesis, cell proliferation, and inflammation^4^. KSHV is among the list of viral pathogens estimated to cause 12–25% of human cancers worldwide^5^.

KSHV has a biphasic life cycle comprised of latent and lytic phases of replication that are distinguished based on divergent gene expression profiles^6^. The dynamics between latent and lytic phases of replication allows the virus to persist for the duration of the host’s lifetime^7^. Notably, KSHV establishes latency in the majority of infected cells^8^; at any given instance, only a subpopulation (<3%) of infected cells display evidence of lytic gene expression^9^. MicroRNAs (miRNAs) are one of the main classes of non-coding RNAs^10^. These are small non-coding RNAs that regulate expression of genes in cells^11^. The human genome encodes thousands of miRNAs^12^. Of late, miRNAs have emerged as a pivotal component of host cell responses to a pathogen including viruses, bacteria, and fungi^13^.

KSHV, human immunodeficiency virus 1 (HIV-1), Epstein-Barr virus (EBV), and herpes simplex virus type 1 (HSV-1) are few examples of the limited number of viruses that encode their own miRNAs^14,15^. KSHV encodes 12 pre-miRNAs which are processed to yield 25 mature miRNAs^16^. The roles of these KSHV-encoded miRNAs is to establish and/or maintain KSHV latency, enhance angiogenesis, spread infected cells, and interfere with the host immune system; all of which are crucial to oncogenesis^17^. Extensive work has been conducted on KSHV encoded miRNAs and the manner by which KSHV replication alters cellular miRNAs^18,19^. However, there is limited work along the lines of understanding the effects of cellular miRNAs in response to early stages of KSHV infection of cells; specifically internalization of the virus. Recently, we employed deep sequencing for the first time, to analyze the miRNA expression profile in KSHV-infected BJAB cells during early stages of infection^20^. In this study, we attempted to decipher how the cellular miRNA-36 (miR-36) alters KSHV infection in physiologically relevant cells: human B, and endothelial cells. We focused on the expression and effects of cellular miR-36 in response to KSHV infection because it was consistently elevated at 15 and 30 min post infection (PI). Our data showed that the over-expression of cellular miR-36 inhibits KSHV infection of cells by dampening expression of interferon induced transmembrane protein 1 (IFITM1). Interestingly, the effect of IFITM1 on the closely related virus, Epstein-Barr virus (EBV) and a distant relative, herpes simplex virus-2 (HSV-2) followed the same pattern as in KSHV. These results reveal a layer of common theme in the regulation of host cell genes by miRNAs in the internalization of KSHV and related viruses.

## Results

### KSHV infection of cells induces host cell miR-36 during early stages of KSHV infection

**Figure 1:**
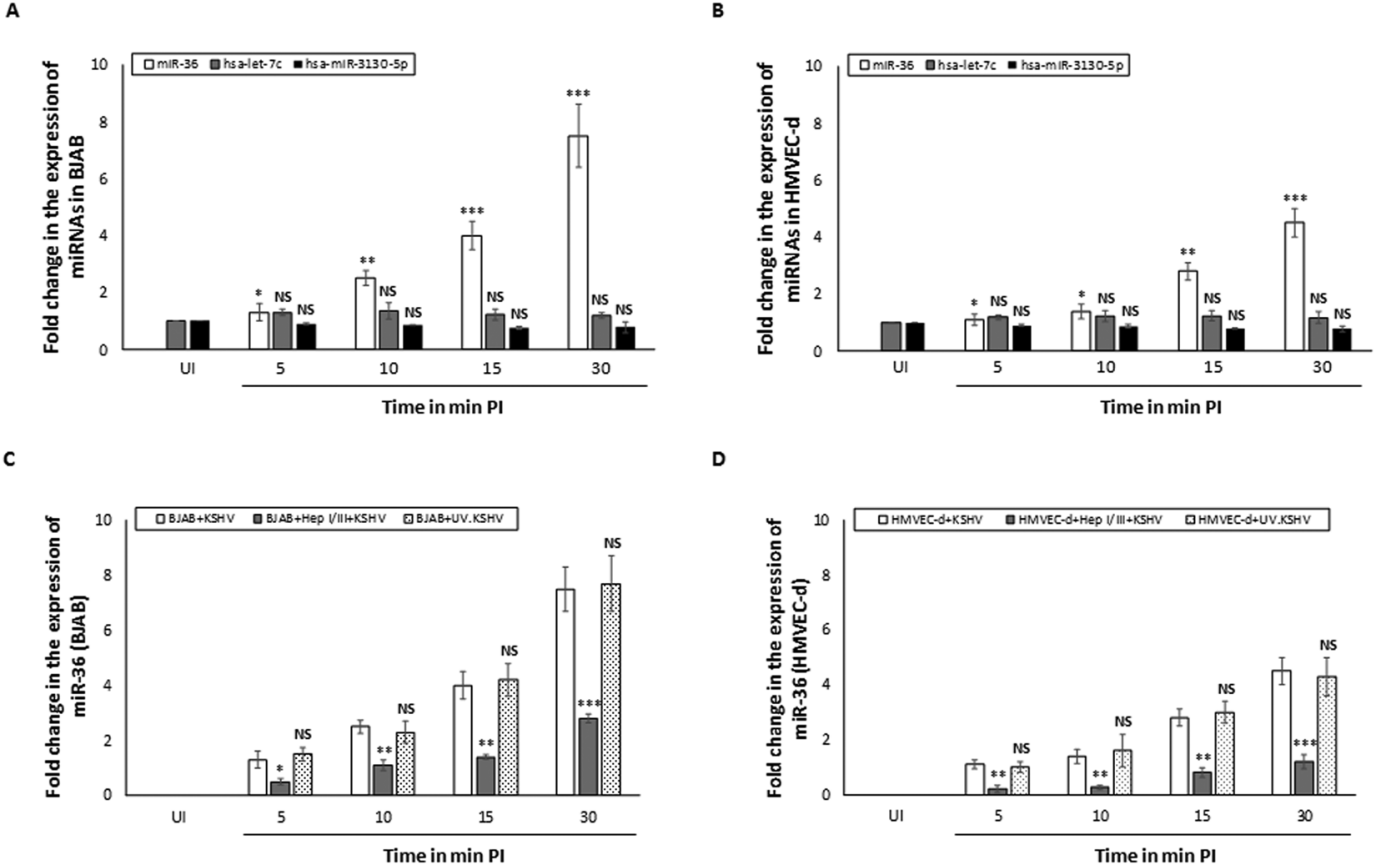
KSHV induces the expression of miR-36 at an early stage of infection. Expression of miR-36, has-let-7c, and has-miR-3130-5p were detected by qRT-PCR at different time points post-infection in cells infected with 10 MOI of KSHV compared to uninfected cells. The qRT-PCR data was plotted for fold changes in the expression of miR-36, has-let-7c and has-miR-3130-5p in (**A**) BJAB cells, and (**B**) HMVEC-d. BJAB (**C**) and HMVEC-d (**D**) cells were either untreated or treated with heparinase I/III prior to performing the infection assay followed by monitoring the expression of miR-36. UV.KSHV was used in this study as a control to understand the importance of an intact virus envelope and associated proteins in inducing miR-36. The relative expression of miRNAs was measured in terms of cycle threshold value (Ct) and normalized to snRNA RNU6B. The *x-axis* indicates the time points post KSHV infection in minutes and *y-axis* indicates fold change in the expression of miR-36. Bars (panels A,B,C,D) represent average ± s.d. of five individual experiments. (**A**,**B**) Student *t* test was performed to compare expression of miR-36 in uninfected cells versus 5, 10, 15, and 30 min PI. In panels ‘C and D’, Student *t* test was performed to study the effect of HS and compare infection of cells with KSHV versus UV.KSHV on miR-36 expression at 5, 10, 15, and 30 min PI. Two-tailed P value of 0.05 or less was considered statistically significant. *p < 0.05; **p,0.01; ***p < 0.001; NS-not significant.

In a recently concluded study, we described a significant increase in the expression of host cell encoded miR-36 as early as 15 min PI of cells^20^. In the present study, we monitored expression of this miR-36 at early time points during KSHV infection of human B and endothelial cells. We employed human B (BJAB) and endothelial (HMVEC-d) cells as they are physiologically relevant cells to KSHV biology. Expression of miR-36 gradually increased from 5 min PI and peaked at 30 min PI in KSHV infected BJAB (Fig. 1A) and HMVEC-d cells (Fig. 1B). Uninfected BJAB and HMVEC-d cells did not express miR-36 (Fig. 1A,B). Expression of known miRNAs, hsa-let-7c and hsa-miR-3130-5p, were not significantly altered when compared to miR-36 during early stages of KSHV infection of cells (Fig. 1A,B). Treatment of cells with 10 units/ml of heparinase I/III for 2 h at 37 °C prior to KSHV infection of cells resulted in a significant drop in the expression of miR-36 in BJAB (Fig. 1C) and HMVEC-d cells (Fig. 1D). Also, infection of both BJAB and HMVEC-d cells with UV.KSHV could induce the expression of miR-36 to comparable levels as the wild-type KSHV (Fig. 1C,D). These results demonstrate KSHV to induce host cell miR-36 very early upon infection. Key features of the novel miR-36 including the secondary structure are provided in Table 1 and supplemental Fig. 1, respectively.

**Table 1:** Characteristics of miR-36.

Name	hsa-miR36
Sequence	CGCAGGAGCCGCGGAGGGCCGGA
Pre-miR36	CGGACTGGCTGGCCGCGCTCTTCGCACGGGGCGCTTTTGCGTGGGGTCGCGCAGGAGCCGCGGAGGGCCGGATCGCT
Chromosome	Chr5
ChromStart	127419226
ChromEnd	127419302
Strand	+
5p Seq	TGGCTGGCCGCGCTCTTCGCAC
3p Seq	CGCAGGAGCCGCGGAGGGCCGGA
Type	Duplex
Hairpin	True

### miR-36 inhibits KSHV infection in BJAB and HMVEC-d cells

**Figure 2:**
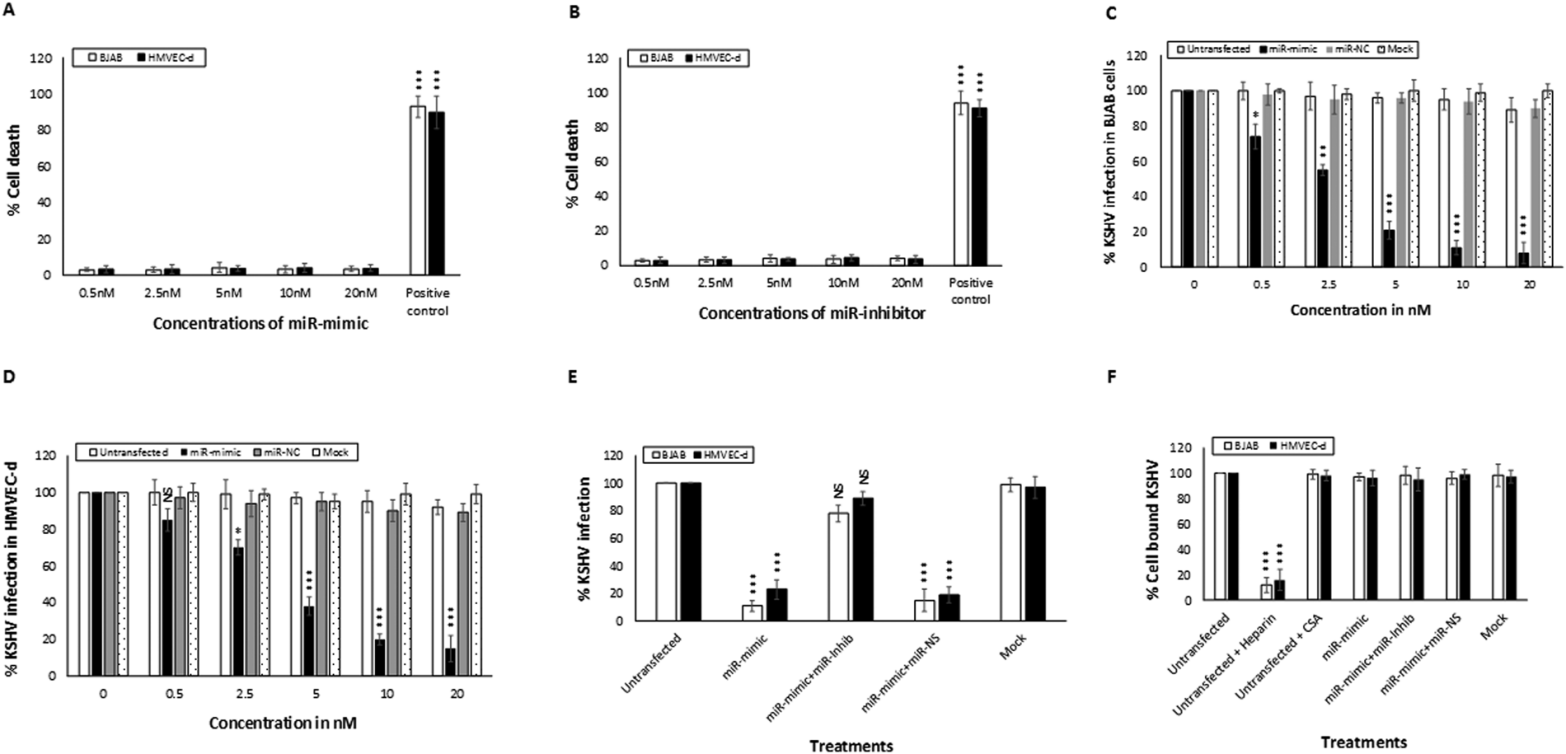
miR-36 overexpression inhibits KSHV infection of cells. To determine the cytotoxic effect of miR-36 mimic and inhibitor, cells were transfected with different concentrations of miR-36 mimic and inhibitor. At 24 h post transfection, lactate dehydrogenase release as an indicator of percentage of cell death was monitored for miR-36 mimic (miR-mimic) (**A**) and miR-inhibitor (**B**). Known inducers of cell death, 1 mg/ml G418 in the case of BJAB cells and 2.5 µg/ml Cytochalasin D for HMVEC-d, were used as positive controls in this study. (**C**,**D**) Transfection of cells with miR-36 mimic specifically inhibit KSHV infection of cells. (**C**) BJAB and (**D**) HMVEC-d cells were either untransfected, mock transfected, or transiently transfected with different concentrations of miR-36 mimic or control mimic (miR-NC) before infection with 10 MOI of KSHV. Data was plotted to represent the percentage of KSHV infection as determined by monitoring the change in KSHV-*ORF50* RNA copy numbers that were detected in transfected cells compared to that detected in untransfected cells (1905 KSHV-*ORF50* RNA copies). Expression of *ORF50* was used as a scale to measure KSHV infection of cells. As reported earlier^29^, the lowest limit of detection in the standard samples was 6–60 copies for the *ORF50* gene. (**E**) Transfection of cells with miR-36 inhibitor opposes the effects of miR-36 mimic on KSHV infection of cells. BJAB and HMVEC-d were either untransfected, mock transfected, transiently transfected with miR-36 mimic, co-transfected with miR-36 mimic and miR-36 inhibitor (miR-mimic + mir-inhib), or co-transfected with miR-36 mimic and nonspecific inhibitor (miR-mimic + mir-NS) before infection with KSHV. Data was plotted to represent the percentage of KSHV infection in transfected cells compared to untransfected cells. The *x-axis* indicates the transfection and *y-axis* indicates the percentage of KSHV infection. (**F**) miR-36 mimic inhibition of KSHV infection of cells is at a post-attachment stage of virus entry. KSHV binding to BJAB and HMVEC-d that were untransfected, mock transfected, transiently transfected with miR-36 mimic, co-transfected with miR-36 mimic and miR-36 inhibitor(miR-mimic + mir-inhib), co-transfected with miR-36 mimic and nonspecific inhibitor (miR-mimic + mir-NS), or untransfected and treated with Heparin or CSA. Data was plotted to represent the percentage of KSHV binding to target cells treated differently compared to the untransfected cells. Bars (panels A–F) represent average ± s.d. of five individual experiments. Student t test was performed to compare groups. Two-tailed P value of 0.05 or less was considered statistically significant. *p < 0.05; **p,0.01; ***p < 0.001; NS-not significant.

**Figure 3:**
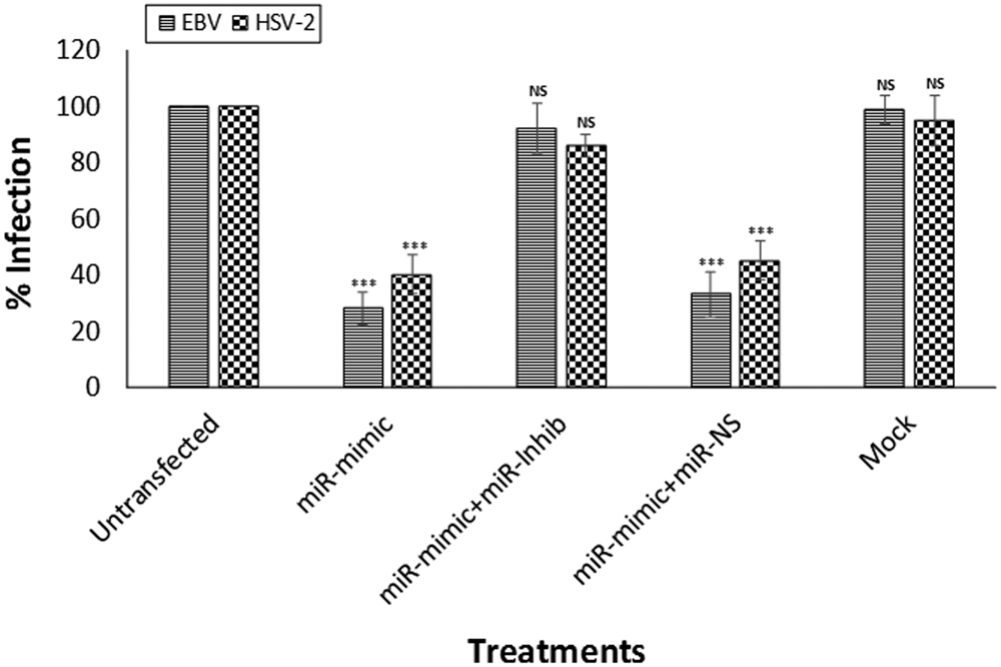
miR-36 overexpression inhibits EBV and HSV-2 infection of BJAB cells. BJAB was either untransfected, mock transfected, transiently transfected with miR-36 mimic (miR-mimic), co-transfected with miR-36 mimic and miR-36 inhibitor (miR-mimic + mir-inhib), or co-transfected with miR-36 mimic and nonspecific inhibitor (miR-mimic + mir-NS) prior to infecting the cells with 10 MOI of EBV or HSV-2. Data was plotted to represent the percentage of virus infection as determined by monitoring the change in RNA copy numbers of EBV-BRLF1 or HSV-2 US1 as detected in transfected cells compared to that detected in untransfected cells (1750 copies of EBV BRLF-1 and 2678 copies of HSV-2 US1). Bars represent average ± s.d. of five individual experiments. Student *t* test was performed to compare viral infection of untransfected cells versus cells transfected with miR-36 mimic, inhibitor, miR-NS, or mock transfected cells. Two-tailed P value of 0.05 or less was considered statistically significant. ***p < 0.001; NS-not significant.

To evaluate the biological effects of miR-36 in target cells, we analyzed the effects of miR-36 mimic and inhibitor on KSHV infection. The range of doses tested in this study is comparable to those reported in the earlier studies^21–25^. The doses of the mimic and inhibitor used in the study did not significantly induce cell death in BJAB and HMVEC-d cells (Fig. 2A,B). Transfection of BJAB (Fig. 2C) and HMVEC-d (Fig. 2D) cells with miR-36 mimic significantly reduced KSHV infection of cells as monitored by the expression of *ORF50* gene as early as 30 min PI. KSHV entry is a quick process and the IE gene, *ORF50*, is expressed immediately upon infection^26^. KSHV infection was not significantly altered in BJAB (Fig. 2C) and HMVEC-d (Fig. 2D) cells that were either transfected with scrambled miRNA control (miR-NC) or mock transfected. Interestingly, the effect of miR-36 mimic on KSHV infection of BJAB and HMVEC-d cells could be significantly reversed by co-transfecting cells with 10 nM of miR-36 inhibitor (Fig. 2E). Co-transfection of cells with miR-NS did not alter the effects of miR-36 mimic (Fig. 2E). To ascertain that the effect of miR-36 mimic was at a post-binding stage of infection, we performed a binding assay. The binding assay performed on BJAB and HMVEC-d cells demonstrated that miR-36 mimic and the miR-36 inhibitor did not block KSHV from binding the target cells (Fig. 2F). Incubating KSHV with heparin but not CSA significantly blocked KSHV from binding cells (Fig. 2F). Our results clearly implicate miR-36 to inhibit KSHV infection of cells. To extend our understanding of the role of miR-36 on other related viruses, we tested the effect of miR-36 mimic and the miR-36 inhibitor on EBV and HSV-2 infection of BJAB cells. Interestingly, miR-36 mimic could significantly block EBV and HSV-2 infection of BJAB cells and this inhibition could be specifically reversed by the miR-inhibitor (Fig. 3).

### miR-36 targets IFITM1

**Figure 4:**
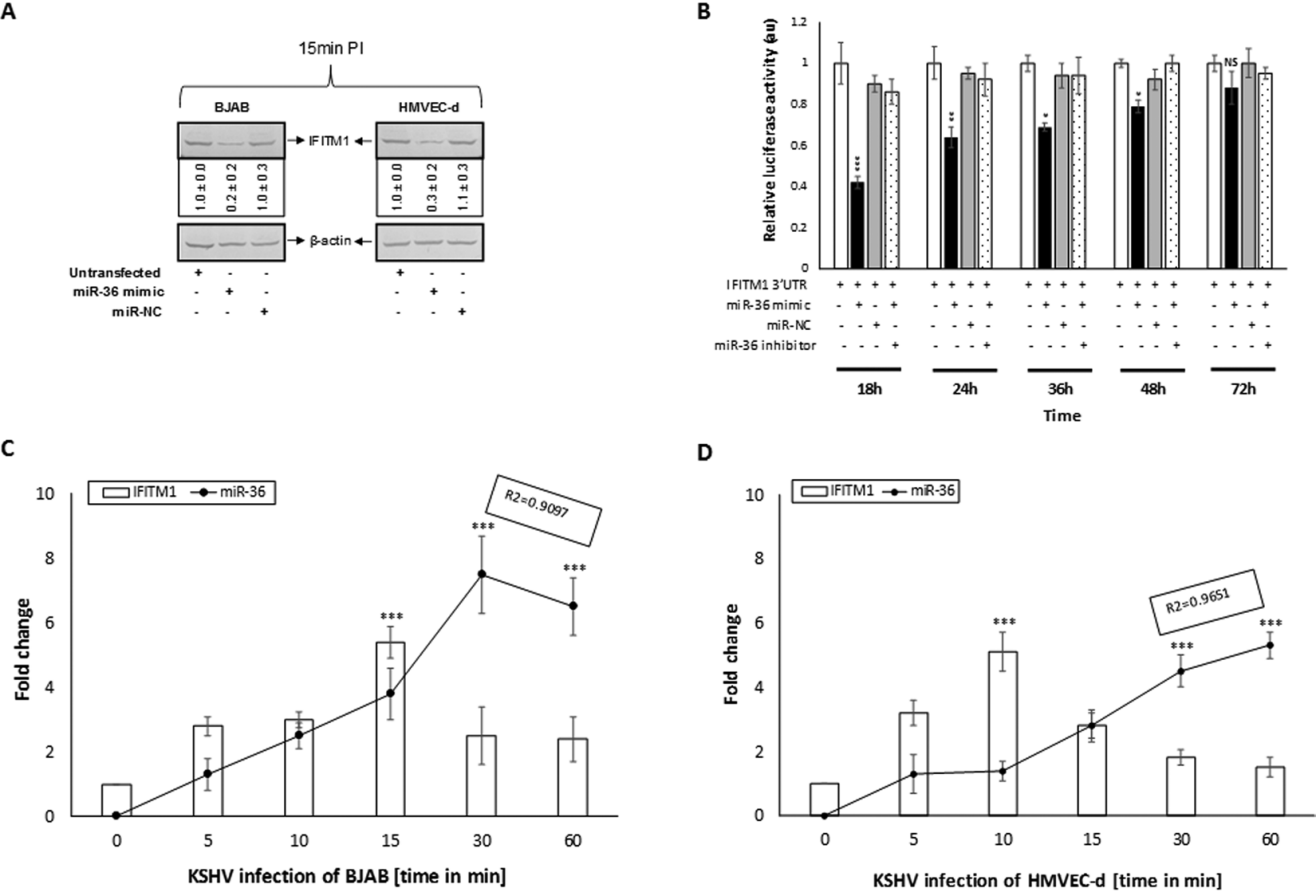
miR-36 targets IFITM1. (**A**) miR-36 mimic lowers KSHV-induced IFITM1 expression. Western blotting analysis demonstrate a decrease in IFITM1 protein levels upon over-expression of miR-36 mimic. BJAB and HMVEC-d cells were either untransfected, transiently transfected with miR-36 mimic, or transfected with control mimic (miR-NC) prior to infecting cells with 10 MOI of KSHV. Monitoring IFITM1 protein levels was performed 15 minutes post KSHV infection and normalized to β-actin protein levels. Data representing the IFITM1 protein expression levels are presented as fold increase (average ± s.d. from three experiments) in the boxes below the panels. The results presented are a representative data and the original full-length blots of the cropped images is provided in Supplemental Fig. 3. (**B**) miR-36 specifically binds and interact with IFITM1. Luciferase activity in 293 cells transfected with Dual-luciferase vector encoding Gaussia Luciferase (GLuc) and secreted alkaline phosphatase (SEAP) with 3′UR of IFITM placed downstream of Glu luciferase reporter (IFITM1 3′UTR). 293 cells were either transfected with IFITM1 3′UTR, co-transfected with IFITM1 3′UTR and miR-36 mimic, co-transfected with IFITM1 3′UTR and control mimic (miR-NC), or co-transfected with IFITM1 3′UTR, miR-36 mimic and miR-36 inhibitor. GLuc activity was monitored at 18 h, 24 h, 36 h, 84, and 72 h post-transfection and was normalized to SEAP. Data is plotted as GLuc/SEAP ratio where the *x-axis* indicates the transfection and time points, and *y-axis* indicates the relative luciferase activity. The relative expression of IFITM1 and miR-36 in KSHV-infected BJAB (**C**) and HMVEC-d (**D**) cells was monitored by qRT-PCR. The expression was measured in terms of cycle threshold value (Ct) and normalized to expression of β-actin and snRNA RNU6B, respectively. The *x-axis* denotes the time point post KSHV infection in minutes and *y-axis* denotes fold change in expression of IFITM1 and miR-36. The R2 values for the miRNA expression during the early course of KSHV infection is provided. Bars (**B**–**D**) represent average ± s.d. of five individual experiments. Student *t* test was performed to compare groups. Two-tailed P value of 0.05 or less was considered statistically significant. *p < 0.05; **p,0.01; ***p < 0.001; NS-not significant.

By using the DIANA and MiRmap tool algorithms, we identified a putative miR-36 binding site located in the 3′-UTR of IFITM1 mRNA (Supplemental Fig. 2). To confirm the ability of miR-36 to specifically inhibit IFITM1 expression, we monitored the expression of IFITM1 in target cells that were untransfected, transfected with miR-36 mimic, or miR-NC prior to infection. Transfection of BJAB and HMVEC-d cells with miR-36 mimic significantly lowered the expression of IFITM1 at 15 min PI compared to untransfected cells and cells transfected with miR-NC (Fig. 4A). Transfection of cells with miR-36 mimic could specifically inhibit IFITM1 expression from 5 min till 48 h PI (data not shown). These results authenticate the fact that IFITM1 expression may well be regulated by miR-36.

In order to determine the bona fide target of miR-36, a luciferase reporter assay was performed. In this assay, two quantifiable genes encoding luciferase proteins were put on a vector. The IFITM1 3′ UTR with the target region was placed downstream GLuc to regulate its translation, and SEAP was placed under no regulation for normalization. 293 cells were co-transfected with the IFITM1 3′ UTR vector plasmid and miR-36 mimic. miR-36 mimic significantly decreased the relative luciferase activity compared to the cells that were transfected with miR-NC (Fig. 4B). In contrast, transfection of cells with miR-inihibitor reversed the ability of miR-36 mimic from lowering the luciferase activity (Fig. 4B). There was an inverse correlation observed in the expression of miR-36 and IFITM1 during the course of KSHV infection of BJAB (Fig. 4C) and HMVEC-d (Fig. 4D) cells. These results suggest that miR-36 directly targets IFITM1 and thereby downregulates its expression.

### IFITM1 expression enhances KSHV, EBV, and HSV-2 infection of cells

**Figure 5:**
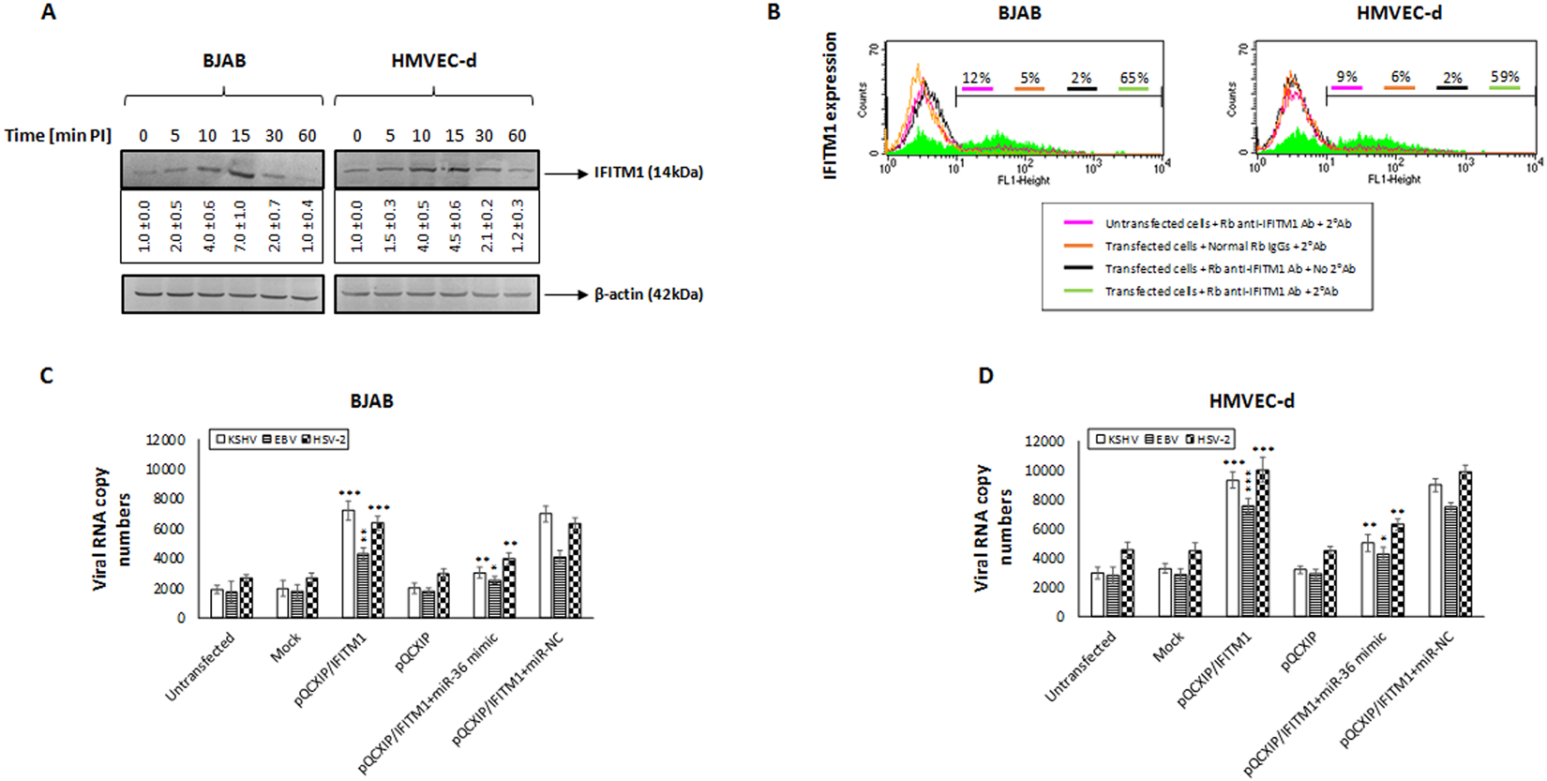
IFITM1 overexpression enhances KSHV infection of cells. (**A**) KSHV infection of cells induce IFITM1 expression. Western blotting analysis demonstrate KSHV infection of target cells to increase IFITM1 protein levels. Expression of IFITM1 levels was normalized to β-actin protein levels. Data representing the IFITM1 protein expression levels are presented as fold increase (average ± s.d. from three experiments) in the boxes below the panels. The results presented are a representative data and the original full-length blots of the cropped images is provided in Supplemental Fig. 4. (**B**) Flow cytometry data confirming the expression of IFITM1 protein in transfected cells. BJAB and HMVEC-d cells transiently transfected with pQCXIP/IFITM1 vector were analyzed for the expression of IFITM1 protein. This was performed by staining untransfected cells (red line) and transfected cells (green line) with rabbit polyclonal anti-IFITM1 antibodies followed by incubation with goat anti-rabbit FITC, before examining by FACS. As a control for the antibodies, transfected cells stained only with polyclonal anti-IFITM1 antibodies (purple dark line) were used. (**C**,**D**) IFITM1 enhances KSHV infection of cells. (**C**) BJAB and (**D**) HMVEC-d cells were untransfected, mock transfected, transiently transfected with pQCXIP/IFITM1, pQCXIP, co-transfected with pQCXIP/IFITM1 and miR-36 mimic, or co-transfected with pQCXIP/IFITM1 and control mimic (miR-NC) prior to infecting with 10 MOI of KSHV, EBV or HSV-2. Data was plotted to represent the percentage of virus infection as determined by monitoring the change in RNA copy numbers of KSHV-*ORF50*, EBV-BRLF1 or HSV-2 US1, respectively. Bars (**C**,**D**) represent average ± s.d. of five individual experiments. Student *t* test was performed to compare the effects of IFITM1 and miR-36 mimic on virus infection of cells compared to appropriate control groups. Two-tailed P value of 0.05 or less was considered statistically significant. **p,0.01; ***p < 0.001; NS-not significant.

IFITM1 protein expression was significantly elevated with KSHV infection of BJAB and HMVEC-d cells (Fig. 5A). The expression of IFITM1 increased in KSHV infected cells as early as 5 min PI which was elevated by 15 min and 10 min PI in BJAB and HMVEC-d cells, respectively, but declined sharply by 30 min PI (Fig. 5A). To confirm a role for IFITM1 in KSHV infection of target cells, we transiently transfected BJAB and HMVEC-d cells with pQCXIP/IFITM1 and the expression of IFITM1 was confirmed by flow cytometry (Fig. 5B). KSHV infection of the above IFITM1 expressing BJAB and HMVEC-d cells was a measure of the expression of *ORF50* at 30 min PI. The idea was to strictly understand the effects of IFITM1 expression on early stages of KSHV infection. BJAB (Fig. 5C) and HMVEC-d (Fig. 5D) cells expressing IFITM1 supported a significantly enhanced KSHV infection compared to those cells that were left untransfected, mock transfected, or transfected with the empty vector. Surprisingly, IFITM1 expression also enhanced HSV-2 and EBV infection of BJAB and HMVEC-d cells (Fig. 5C,D). Interestingly, co-transfection of the above cells with miR-36 mimic could significantly drop KSHV infection of cells compared to miR-NC (Fig. 5C,D).

**Figure 6:**
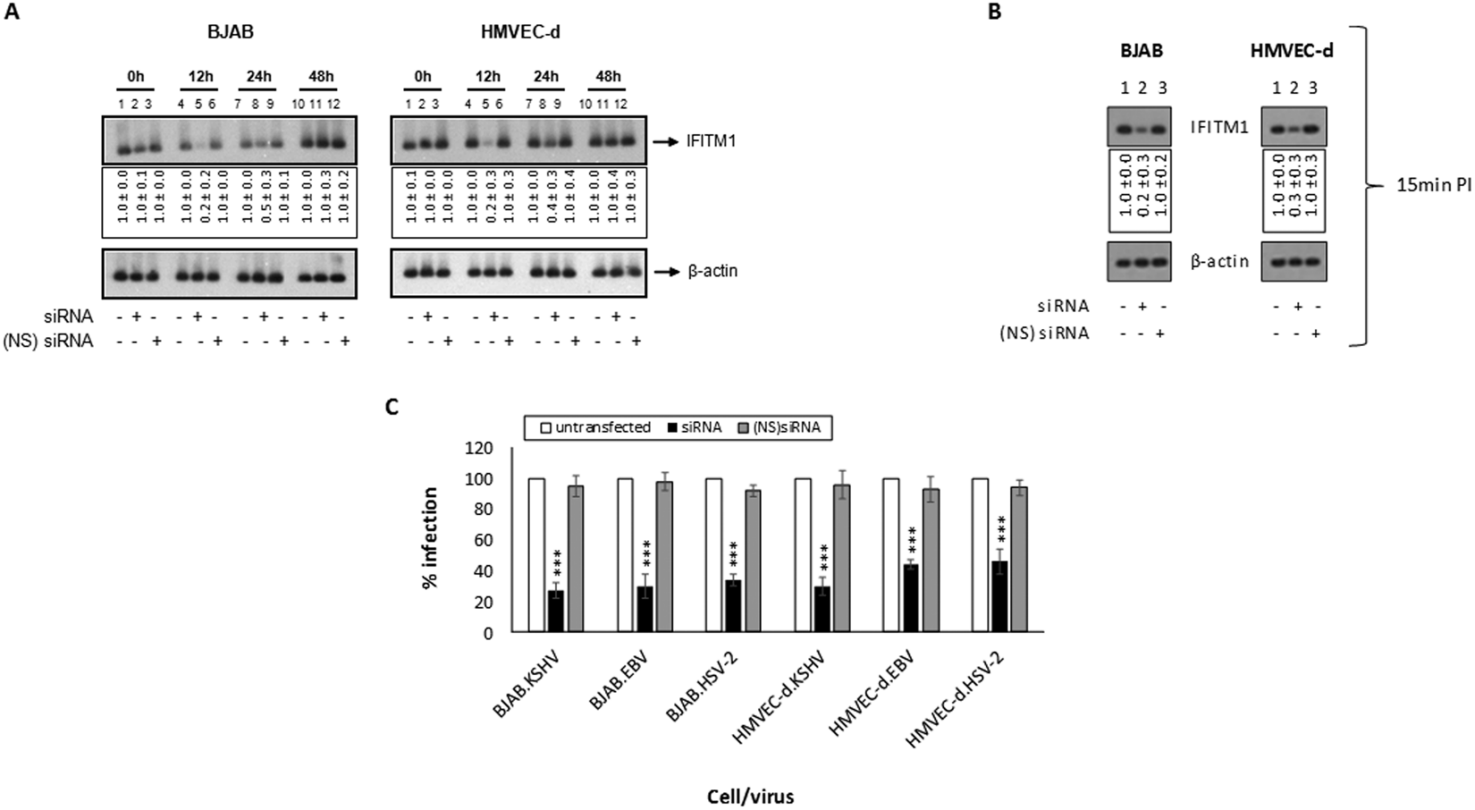
Knock-down of IFITM1 by siRNA decreases KSHV, EBV and HSV-2 infection of cells. (**A**) Northern blotting to monitor the effect of transfecting cells with siRNA specific to IFITM1. Target cells were untransfected or transfected either with ds siRNA or (NS)siRNA controls. After 0, 12, 24, and 48 hours after transfection, total RNAwas isolated from the cells and subjected to Northern blotting as per standard protocols to monitor IFITM1and β-actin mRNA. Data representing the IFITM1 mRNA expression levels are presented as fold increase (average ± s.d. from three experiments) in the boxes below the panels. The results presented are a representative data and the original full-length blots of the cropped images is provided in Supplemental Fig. 5. (**B**) BJAB and HMVEC-d cells were un-transfected, transfected with IFITM1-specific siRNA, or NS- siRNA prior to infecting cells with 10 MOI of KSHV. IFITM1 mRNA levels was monitored at 15 min PI by Northern blotting and normalized to β-actin levels. (**C**) KSHV, EBV, and HSV-2 infection significantly lowered in cells silenced for IFITM1 expression. BJAB and HMVEC-d were either un-transfected, transfected with IFITM1-specific siRNA, or transfected with (NS) siRNA before infecting with KSHV, EBV or HSV-2. Data was plotted to represent the percentage of virus infection as determined by monitoring the change in RNA copy numbers of KSHV-*ORF50*, EBV-BRLF1, or HSV-2 US1. Bars represent average ± s.d. of five individual experiments. Student t test was performed to compare groups. Two-tailed P value of 0.05 or less was considered statistically significant. ***p < 0.001.

To further confirm the role of IFITM1 in KSHV infection of cells, we first transfected cells with siRNA specific for IFITM1. Northern blotting was performed at 0, 12, 24, and 48 hours after transfection as per the standard protocols to monitor IFITM1 mRNA expression (Fig. 6A). The levels of IFITM1 mRNA was significantly suppressed in BJAB and HMVEC-d cells by siRNA when compared with a (NS)siRNA control (Fig. 6A). A IFITM1 mRNA inhibition of 82% ± 7%, 74% ± 9%, and 45% ± 6% was observed in BJAB cells at 12, 24, and 48 hours, respectively, after siRNA transfection (Fig. 6A). A IFITM1 mRNA inhibition of 78% ± 9%, 65% ± 7%, and 34% ± 7% was observed in HMVEC-d cells at 12, 24, and 48 hours, respectively, after siRNA transfection. IFITM1 expression levels were not significantly altered by the (NS)siRNA controls in both the cells tested, demonstrating the specificity of the siRNA used (Fig. 6A). IFITM1 expression in target cells transfected with siRNA specific to IFITM1 was significantly lowered at 15 min post KSHV infection (Fig. 6B). In contrast, KSHV induced IFITM1 expression in untransfected or cells transfected with (NS)siRNA were not altered (Fig. 6B). On the same lines, KSHV infection in cells silenced for the expression of IFITM1 was significantly lower compared to cells that were untransfected or transfected with (NS)siRNA (Fig. 6C). Silencing the expression of IFITM1 also decreased EBV and HSV-2 infection of the above cells (Fig. 6C). The above viral infections were monitored by performing *qRT-PCR*. Taken together, the results clearly implicate a role for IFITM1 in enhancing KSHV, EBV, and HSV-2 infection of cells.

## Discussion

Since miRNAs discovery over 20 years ago, miRNAs have been established as key players in the molecular mechanisms of mammalian biology including the maintenance of normal homeostasis and the regulation of disease pathogenesis. Host miRNAs also play a crucial role in mounting an immune response against microbial infections including those caused by viruses^27^. Several viruses belonging to herpesvirus, polyomavirus, hepadnavirus, adenovirus, and retrovirus encode miRNAs^14,15^. Virus-encoded miRNAs (vmiRNA) identified in virus-infected cells significantly influence viral replication and disease progression by modulating viral as well as host cellular mRNA. Many of the cellular miRNAs affect viral replication either directly by binding to the viral genome or indirectly by targeting host factors related to replication^28^. There are several published works that describe virus and cellular encoded miRNAs in KSHV pathogenesis^10,18^. However, to date, there is no report on the role of cellular miRNAs during early stages of KSHV infection.

Recent studies from our laboratory using a combination of deep sequencing and qRT-PCR identified cellular miR-36 to activate as early as 15 min PI^20^. In the current studies, we employed physiologically relevant human B cells (BJAB) and endothelial cells (HMVEC-d) to make the study more meaningful to KSHV biology. Accordingly, using specific primers we analyzed the expression profile of cellular miR-36 during the first 30 min of KSHV infection of cells. A successful viral entry was a measure of KSHV to enter cells and express immediate early gene, *ORF50*. The expression of *ORF50* was monitored by qRT-PCR^26,29^. KSHV infection of BJAB and HMVEC-d cells rapidly triggered the expression of miR-36 as early as 5 min PI that peaked at 30 min PI (Fig. 1). Expression of miR-36 in KSHV infected BJAB is higher than those in HMVEC cells. This could be attributed to the inherited variations observed between different cells in the manner by which they respond to virus infection^30^. Moreover, the expression level of miRNAs has been shown to vary among tissues, cell types, and even between cells of the same lineage^31,32^.

Cellular miR-36 is triggered by UV.KSHV infection of cells while treating target cells with heparinase I/III prior to infecting cells with the wild-type KSHV failed to induce expression of miR-36. Treatment of cells with heparinase I/III cleave heparan sulfate (HS) at different sites and liberate them from the cell surface^33^. KSHV binds to a target cell via interacting with HS expressed on the cell surface^34^. Taken together, our results indicate the following: (i) it is the interactions between the virus envelope proteins and the host cells that trigger miR-36 response; and (ii) binding of KSHV to cells is critical to miR-36 expression in cells. miR-36 mimic specifically inhibited KSHV infection of BJAB and HMVEC-d cells (Fig. 2C,D). Transfection of cells with miR-36 inhibitor reversed the effects of miR-36 mimic on KSHV infection of cells (Fig. 2E). The effects of miR-36 mimic and inhibitor was specific as the scramble negative control (miR-NC) and non-specific inhibitor (miR-NS) did not significantly alter KSHV infection of cells (Fig. 2C,D,E). It was concluded that the effects of miR-36 mimic and inhibitor on KSHV infection was at a post attachment stage of internalization as they did not adversely affect virus binding to cells (Fig. 2F).

In this study, we originally wanted to use two relevant viruses as negative controls to better understand the specificity of miR-36 on KSHV infection of cells. EBV and HSV-2 were selected as controls: EBV, like KSHV, belongs to γ-herpesvirinae while HSV-2 belongs to *α-herpesvirinae*. Interestingly, we observed a similar effect of miR-36 mimic and inhibitor on EBV and HSV-2 infection of cells (Fig. 2F). This could be due to the fact that *α, β, and γ-herpesviruses* exhibit and share a common three-dimensional capsid structure along with the fact that there is quite a bit of homology in the glycoproteins being expressed on the viral envelope^35^.

One miRNA may regulate many genes as its targets, while one gene may also be targeted by many miRNAs^36^. Accordingly, miR-36 can possibly target several genes (Supplemental Fig. 6). Using bioinformatics tools, we identified IFITM1 to be the most promising targets to miR-36. IFITM is a member of the interferon-induced 125–133 amino acid protein family including IFITM1, IFITM2, IFITM3, IFITM5 and IFITM10. This family of proteins is located on chromosome 11 of the human genome and originally described as highly inducible genes by α- and γ-interferons (IFNs)^37,38^. The three members of the IFITM proteins (IFITM1, IFITM2, and IFITM3) have gained prominence as novel antiviral IFN-stimulated genes (ISGs)^39^. Hence, we set out to test the effect of transfecting target cells with IFITM1-3 genes on KSHV infection of target cells. Over-expressing IFITM1 significantly enhanced KSHV infection of cells; IFITM3 moderately enhanced KSHV infection while IFITM2 did not alter the viral infection (Supplemental Fig. 7). Based on these results, we focused our further studies on IFITM1 in terms of miR-36 and early stages of KSHV infection. Using bioinformatic tools it was determined that the miR-36 target IFITM1 expression. Luciferase assay demonstrated the ability of miR-36 to physically interact with IFITM1 (Fig. 4B). miR-36 mimic specifically inhibited IFITM1 expression that could be reverted by transfecting cells with miR-36 inhibitor (Fig. 4B). There was an apparent inverse correlation observed between the KSHV-induced IFITM1 expression and miR-36 response (Fig. 4C,D). We propose the sharp decline in the expression of IFITM1 30 min PI is because of an increase in the expression of miR-36 (Fig. 4C,D). Taken together, our study established a direct association between virus-induced IFITM1 and endogenous miR-36 expression in the biology of KSHV.

IFITM1 is expressed in many cell types including leukocytes and endothelial cells^37,40^. IFITM1 modulates cell functions including immunological responses, cell proliferation, cell adhesion, and germ cell maturation^41^. As other IFITM proteins, IFITM1 is significantly upregulated by interferons type I and II and is critical for anti-virus response of innate immunity^42^. Recent reports indicate IFITM1 to play a significant role in virus entry. IFITM1 inhibits entry of many RNA viruses including influenza A H1N1 Virus, West Nile Virus, Dengue Virus, HIV, and HCV^42–44^. The suggested mechanisms by which IFITM proteins restrict the above virus infections include inhibition of virus binding to corresponding cellular receptors, inhibition of endocytosis, and acting as pattern recognition receptors by sensing virus infection and activation of downstream cellular signaling pathways^42^. These proteins inhibit fusion of viral membranes with cellular endosomal vesicular membranes by blocking the creation of hemifusion, reducing membrane fluidity and curvature, and by possibly disrupting intracellular cholesterol homeostasis^45,46^. However, IFITMs can also enhance viral infection of cells: (i) both IFITM1 and IFITM3 modestly enhanced human papillomavirus 16 (HPV-16) infection of a variety of cells^47^; and (ii) Zhao *et al*. have shown type I IFN-α, IFN-γ, and type II IFN-λ to significantly promote infection of human coronavirus, HCoV-OC43 by the induction of IFITM proteins. The authors reported that the over-expression of IFITM3 significantly increased susceptibility of Huh7.5 cells to HCoV-OC43 infection^48^. In general, the IFITM family of proteins affects virus entry of cells. Over-expression of IFITM1 significantly enhanced KSHV infection of target cells (Fig. 5C,D) while silencing expression of IFITM1 had the opposite effect (Fig. 6). More interestingly, we observed identical effects of IFITM1 in enhancing EBV and HSV-2 infection of cells (Fig. 6C).

IFNs are generally considered to be antiviral cytokines that inhibit virus infection of cells by stimulating ISGs^49^. In fact, we observed a significant increase in the expression of IFN-α and -γ during the early stages of KSHV infection of BJAB and HMVEC-d cells (Supplemental Fig. 8). If that is the case, how can IFITM1 enhance infection of not just KSHV; but also of EBV and HSV-2? Such a scenario can be possible only if the virus infection is not altered by IFNs and associated proteins^48^. Interestingly enough, herpesviruses as a group (including KSHV) is considered to be relatively insensitive to the antiviral effects of IFNs in a variety of different cell systems^50,51^. In a way, our results demonstrate for the first time that herpesviruses, KSHV, EBV, and HSV-2, not only hijack IFITM1 to their benefit in facilitating virus entry into cells but also evade the IFN-induced antiviral effects.

This study provides a new insight to virus infection. KSHV (including EBV and HSV-2) interactions with target cells induce IFITM1 within minutes, which facilitate virus entry into cells. KSHV-induced IFITM1 is part of the innate immune activation system that occurs in an antigen-independent fashion^52^ and relies on the ability of the host to recognize virus via specific pattern recognition receptors^53^. To counter the effects of KSHV-induced IFITM1, infected cells respond within a short period of time by inducing expression of miR-36. Cellular miR-36 in turn inhibits expression of IFITM1 and thus limit virus infection of cells. Perhaps, this could be a mechanism of superinfection resistance (SIR)^54^ developed by cells towards KSHV and other related viral pathogens. IFITM1 suppression by miR-36 may have direct and indirect effects of KSHV pathobiology: (i) directly limit KSHV infection of cells; (ii) block cell proliferation/division^55^ and thereby promote KSHV latency^56^; and (iii) reduce virus-induced inflammation. Such effects of IFITM1 on the biology of KSHV will be better understood by employing the three-dimensional (3-D) cell culture models as they mimic certain aspects of the tissue environment^57,58^. For all this time, studies on virus entry have always focused primarily on the roles of virus encoded glycoproteins and their cognate host cell receptors. To our knowledge, this is the first report of a miRNA influencing KSHV infection of cells and this, we hope, will open doors to a further understanding of virus entry; after all, it is the miRNAs that regulate the gene function.

Of late, miRNA-based therapeutics have been used to effectively treat autoimmune diseases^59^, and cancers including prostate cancer, and leukemia^60^ in animal models. The fact that miRNAs can influence virus entry is fascinating as miRNA-based therapeutics like the use of miR-mimics can effectively be used to treat virus entry including pathogenesis. There are several questions that need to be answered and they are as follows: (i) How does IFITM1 enhance KSHV infection of cells? Does it physically bind KSHV envelope-associated protein and facilitate endocytosis? (ii) What is the role of host cell receptors in the IFITM1-facilitated virus entry? (iii) What is the dynamics between the expression pattern of IFITM1 and 3 in regulating KSHV infection of cells? And (iv) Can induction of miR-36 by KSHV infection prevent infected cells from being superinfected with KSHV and other related herpesviruses, *in vivo*? Current studies in our lab are dedicated to answer these questions.

## Materials and Methods

### Cells

Human Burkitt lymphoma B cell line (BJAB), human foreskin fibroblasts (HFFs; Clonetics,Walkersville, MD), HEK-293 (293), and dermal microvascular endothelial cells (HMVEC-ds; CC-2543; Clonetics) were used in this study. BJAB cells were propagated in phenol red–free RPMI medium (Invitrogen, Carlsbad, CA) while HFF and 293 cells were cultured in Dulbecco modified Eagle medium (DMEM) containing 10% charcoal-stripped fetal bovine serum (FBS; Atlanta Biologicals, Lawrenceville, GA), L-glutamine, and antibiotics^61^. HMVEC-d cells were propagated in EGM MV-microvascular endothelial cell medium (Clonetics) as per standard protocols. The passage numbers for HFFs and HMVEC-ds used in this study ranged between 6–10, and 5–9, respectively. All the cells used in this study were negative for mycoplasma as tested by *Mycoplasma* PCR ELISA, Roche Life Science. For culture conditions, refer to supplemental data.

### Virus

The viruses used in this study were wild-type KSHV^26^, herpes simplexvirus-2 (HSV-2)^62^, and Epstein-Barr virus (EBV)^63^. We generated ultraviolet (UV) inactivated KSHV (UV.KSHV) as per early studies^20^.

### Cytotoxicity assay

The LDH assay was performed using the CytoTox 96 non-radioactive kit (Promega) as per earlier studies^26^. Target cells were treated with different concentrations of miR-36 mimic and inhibitor at 37 °C in a V-bottom 96-well plate. After a 24 h incubation, the cells were analyzed for the expression of LDH, as an indicator of cell death. The LDH assay was performed using the CytoTox 96 non-radioactive kit (Promega) as per earlier studies^26^. G418 (Sigma-Aldridge, St. Louis, MO) and cytochalasin D (Sigma-Aldridge) were used as known cell death inducers.

### Virus infection of cells, RNA extraction, and monitoring virus infection

BJAB, HFF, and HMVEC-d cells were infected with 10 multiplicity of infection (MOI)^29^ of KSHV, EBV, and HSV-2. The cells were left uninfected or infected for 5, 10, 15, and 30 min prior to washing the cells twice in PBS and processed appropriately for RNA extraction. Total RNA was extracted using TRIzol (Invitrogen, Carlsbad, CA). The RNA concentration was measured with a NanoDrop ND-2000 spectrophotometer (Thermo Fisher Scientific, Waltham, MA), and then verified for quality using an Agilent 2100 Bioanalyzer (Agilent Technologies, Santa Clara, CA). Only the RNA samples with 260/280 ratios of 1.8 to 2.0 were used in the study.

Extracted RNA was used to synthesize cDNA and the expression of *ORF50* was monitored by qRT-PCR using specific primers as per earlier studies^26^ Expression of *ORF50* was used as a scale to measure KSHV infection of cells. As reported earlier^29^, the lowest limit of detection in the standard samples was 6–60 copies for the *ORF50* gene. The results from the use of *ORF50* primers were consistently confirmed by monitoring the expression of another viral immediate early (IE) gene, vGPCR (data not shown). EBV and HSV-2 infection was monitored using specific primers to BRLF1 (homolog of KSHV *ORF50*)^64^ and HSV-2 IE gene, US1^65^.

### Transfections of cDNA and miRNA

Target cells were transiently transfected with plasmid DNA using FuGene HD (Promega, Madison, WI) as per manufacturer’s recommendations. The plasmid, pQCXIP encoding IFITM1, used in this study was kindly gifted to us by Dr. Michael Farzan (The Scripps Research Institute, Jupiter, USA). FuGene HD/DNA ratios of 3:1 for adherent cell lines and 6:1 for suspension cell lines were used. Transfection of target cells with different concentrations of miR-36 mimic, or scramble control (miR-NC); miRNA inhibitor to miR-36 and non-specific (NS) inhibitor (Sigma-Aldridge) was achieved using FuGene HD reagent as per standard laboratory procedures. These were transient transfections and experiments using these cells were conducted 48 h post transfection.

### Binding assay

The effect of miR-mimic and inhibitor on KSHV binding to target cells was assessed by PCR detecting the cell-bound KSHV DNA. Briefly, untransfected cells or cells transfected with miR-mimic or inhibitor were infected with 10 MOI of KSHV at +4 °C. After 60 min of incubation with virus, cells were washed three times with PBS to remove the unbound virus. Cells were pelleted, and total DNA including those representing the cell bound KSHV was isolated using DNeasy kit (Qiagen, Valencia, CA) and subjected to qPCR analysis monitoring *ORF50* according to recently published work^29^. Incubating KSHV with 100 µg/ml of heparin and chondroitin sulfate A (CSA; Sigma-Aldridge) for 1 h at 37 °C were used as known positive and negative controls.

### Flow cytometry

Flow cytometry was used to monitor expression of IFITM1 in the cells as per earlier protocols^29^. Briefly, target cells were fixed in 10 ml of ice-cold acetone for 20 min, washed thrice in PBS prior to incubating cells in 25 µg/ml of rabbit polyclonal antibody to IFITM1 (EMD Millipore, Billerica, MA) for 60 min at 4 °C. The cells were washed thrice in PBS and further incubated with FITC conjugated appropriate secondary IgG at 4 °C for 30 min, washed and analyzed in a FACScan flow cytometer (Becton Dickinson) with appropriate gating parameters.

### Western blotting

All the buffers used in this project were made with water that was endotoxin and pyrogen free. Western blotting was conducted as per earlier studies^29^ using the following primary antibodies: rabbit anti-IFITM1 polyclonal antibody (EMD Millipore) and mouse anti-actin antibodies (Clone AC-74; Sigma-Aldridge).

### Real-time *qRT-PCR* analysis of the expression of miRNAs and IFNs

The quality of RNA was tested using a spectrophotometer. Only the RNA samples with 260/280 ratios of 1.8 to 2.0 were used in the study. Approximately 500 ng of RNA was reverse transcribed in a 25 µl reaction volume using the All-in-one^TM^ miRNA qRT-PCR detection kit (GeneCopoeia, Rockville, MD). Briefly, the cDNA was synthesized in a 25 μl reaction mix containing 5 μl of 5x reaction buffer, 2.5 U/μl Poly A Polymerase, 10 ng/μl MS2 RNA, and 1 µl RTase Mix. The reaction was performed at 37 °C for 60 min and terminated at 85 °C for 5 min. cDNA that was produced in the RT reaction was diluted ten-fold and was used as the template for the PCR reaction in an Applied Biosystems ViiA 7 Real-Time PCR System (Life Technologies, USA). In this system, MS2 RNA was used as an external reference for the quality of the extracted miRNAs, and RNU6B, RNU44, RNU48, and RNU49 were used for normalization. The expression levels of miRNAs were measured employing qRT-PCR with the SYBR green detection and specific forward primer for the mature miRNA sequence and the universal adaptor reverse primer (GeneCopoeia, USA). Expression of IFN-α, -β, and -γ by qRT-PCR was conducted as per earlier protocols^26^ using appropriate primers^66–68^.

### Dual-luciferase reporter assay

Luciferase reporter plasmids with wild-type IFITM1 3′-UTR were purchased from GeneCopoeia (Rockville, MD). 293 cells were plated onto 6-well plates. At 24 h post-plating, 293 cells were co-transfected with IFITM1 3′-UTR luciferase reporter plasmid and miR-36 mimic or miR-NCNA scramble control (miR-NC) using FuGene HD (Promega). At 12, 24, 48 h post transfection, supernatants were collected from each treatment and the luciferase activity measured using the Secrete-Pair Dual Luminescence Assay Kit (GeneCopoeia) as per the manufacturers’ recommendations.

### Northern blotting

Northern blotting to monitor IFITM1 and β-actin expression was performed using a DIG Luminescent Detection Kit (Roche, Indianapolis, IN) as per the manufacturer’s recommendations^26^.

### Silencing IFITM1 using siRNA

Expression of IFITM1 was inhibited by the transfection of double-stranded (ds) RNA oligos as per standard protocols^26^. IFITM1 siRNA was purchased from Dharmacon RNA Technologies (Lafayette, CO). Briefly, 1 × 10^6^ cells were washed twice in RPMI and incubated in phenol red–free RPMI supplemented with 5% FBS at 37 °C. After 24 hours incubation (considered as 0 h for experiments in Fig. 6A), the target cells were transfected with either ds short interfering RNAs (siRNAs) or the nonspecific (NS) controls using Fugene HD as per manufacturer’s recommendations (Promega). At 0, 12, 24, and 48 hours after transfection, total RNA was isolated from the cells and subjected to Northern blotting to monitor the expression of IFITM1 and β-actin mRNA as per the protocol mentioned in the “Materials and methods” section describing Northern blotting. In another set of experiments, untransfected cells and cells transfected with siRNA or (NS) siRNA for 12 h were infected with 10 MOI of KSHV. At the end of 30 min PI, KSHV infection was assessed by monitoring ORF50 expression by qRT-PCR.

### Data Availability Statement

All data generated or analyzed during this study are included in this published article (and its Supplementary Information files).

## Electronic supplementary material

Supplementary Information
