# Supplementary material for: miRNA-36 inhibits KSHV, EBV, HSV-2 infection of cells via stifling expression of interferon induced transmembrane protein 1 (IFITM1)
Source: Sci Rep. 2017 Dec 21;7:17972. doi: 10.1038/s41598-017-18225-w (PMC5740118; doi:10.1038/s41598-017-18225-w)
Supplement: Supplementary file 1 — Supplementary Information [file 41598_2017_18225_MOESM1_ESM.pdf]

miRNA-36 inhibits KSHV, EBV, HSV-2 infection of cells via stifling expression of interferon induced transmembrane protein 1 (IFITM1)

Hosni A.M. Hussein & Shaw M. Akula*
Department of Microbiology & Immunology, Brody School of Medicine at East Carolina University, Greenville, NC 27834.

Short title: miRNA-36 inhibits KSHV infection of human B cells

Word count for text: 3517

Word count for abstract: 189

Figure: 6

Table: 1

Reference count: 68

Supplemental figures: 8

Address correspondence to: Shaw M. Akula, Department of Microbiology & Immunology, Brody School of Medicine, East Carolina University, Greenville, North Carolina, USA 27834. Phone: (252)744-2702; Fax: (252) 744-3104; Email: akulas@ecu.edu

SUPPLEMENTAL DATA

1. MATERIALS AND METHODS

Cell culture: BJAB cells were propagated in phenol red–free RPMI medium (Invitrogen, Carlsbad, CA) while HFF and 293 cells were cultured in Dulbecco modified Eagle medium (DMEM) containing 10% charcoal-stripped fetal bovine serum (FBS; Atlanta Biologicals, Lawrenceville, GA), L-glutamine, and antibiotics[80]. HMVEC-d cells were propagated in EGM MV-microvascular endothelial cell medium (Clonetics) as per standard protocols. The passage numbers for HFFs and HMVEC-ds used in this study ranged between 6 - 10, and 5 - 9, respectively[42,80].

Cytotoxicity assay

Target cells were treated with different concentrations of miR-36 mimic and inhibitor at 37°C in a V-bottom 96-well plate. After a 24h incubation, the cells were analyzed for the expression of LDH, as an indicator of cell death. The LDH assay was performed using the CytoTox 96 non-radioactive kit (Promega) as per earlier studies[33]. G418 (Sigma-Aldridge, St. Louis, MO) and cytochalasin D (Sigma-Aldridge) were used as known cell death inducers.

Virus infection of cells, RNA extraction, and monitoring virus infection

BJAB, HFF, and HMVEC-d cells were infected with 10 multiplicity of infection (MOI)[42,79] of KSHV, EBV, and HSV-2. The cells were left uninfected or infected for 5, 10, 15, and 30min prior to washing the cells twice in PBS and processed appropriately for RNA extraction. Total RNA was extracted using TRIzol (Invitrogen, Carlsbad, CA). The RNA concentration was

measured with a NanoDrop ND-2000 spectrophotometer (Thermo Fisher Scientific, Waltham, MA), and then verified for quality using an Agilent 2100 Bioanalyzer (Agilent Technologies, Santa Clara, CA). Only the RNA samples with 260/280 ratios of 1.8 to 2.0 were used in the study.

Extracted RNA was used to synthesize cDNA and the expression of *ORF50* was monitored by qRT-PCR using specific primers as per earlier studies[33]. Expression of *ORF50* was used as a scale to measure KSHV infection of cells. As reported earlier[42], the lowest limit of detection in the standard samples was 6–60 copies for the *ORF50* gene. The results from the use of *ORF50* primers were consistently confirmed by monitoring the expression of another viral immediate early (IE) gene, vGPCR (data not shown). EBV and HSV-2 infection was monitored using specific primers to BRLF1 (homolog of KSHV *ORF50*)[81] and HSV-2 IE gene, US1[82].

Flow cytometry

Flow cytometry was used to monitor expression of IFITM1 in the cells. Briefly, target cells were fixed in 10ml of ice-cold acetone for 20min, washed thrice in PBS prior to incubating cells in 25µg/ml of rabbit polyclonal antibody to IFITM1 (EMD Millipore, Billerica, MA) for 60min at 4°C. The cells were washed thrice in PBS and further incubated with FITC conjugated appropriate secondary IgG at 4°C for 30 min, washed and analyzed in a FACScan flow cytometer (Becton Dickinson) with appropriate gating parameters.

Real-time *qRT-PCR* analysis of the expression of miRNAs

The quality of RNA was tested using a spectrophotometer. Only the RNA samples with 260/280 ratios of 1.8 to 2.0 were used in the study. Approximately 500ng of RNA was reverse transcribed in a 25µl reaction volume using the All-in-one™ miRNA qRT-PCR detection kit (GeneCopoeia,

Rockville, MD). Briefly, the cDNA was synthesized in a 25μl reaction mix containing 5μl of 5x reaction buffer, 2.5U/μl Poly A Polymerase, 10ng/μl MS2 RNA, and 1μl RTase Mix. The reaction was performed at 37°C for 60 min and terminated at 85°C for 5 min. cDNA that was produced in the RT reaction was diluted ten-fold and was used as the template for the PCR reaction in an Applied Biosystems ViiA 7 Real-Time PCR System (Life Technologies, USA). In this system, MS2 RNA was used as an external reference for the quality of the extracted miRNAs, and RNU6B, RNU44, RNU48, and RNU49 were used for normalization. The expression levels of miRNAs were measured employing qRT-PCR with the SYBR green detection and specific forward primer for the mature miRNA sequence and the universal adaptor reverse primer (GeneCopoeia, USA).

Silencing IFITM1 using siRNA

Expression of IFITM1 was inhibited by the transfection of double-stranded (ds) RNA oligos as per standard protocols[80]. IFITM1 siRNA was purchased from Dharmacon RNA Technologies (Lafayette, CO). Briefly, 1 x 10^6 cells were washed twice in RPMI and incubated in phenol red–free RPMI supplemented with 5% FBS at 37°C. After 24 hours incubation (considered as 0h for experiments in **Fig. 6A**), the target cells were transfected with either ds short interfering RNAs (siRNAs) or the nonspecific (NS) controls using Fugene HD as per manufacturer's recommendations (Promega). At 0, 12, 24, and 48 hours after transfection, total RNA was isolated from the cells and subjected to Northern blotting to monitor the expression of IFITM1 and β-actin mRNA as per the protocol mentioned in the "Materials and methods" section describing Northern blotting. In another set of experiments, untransfected cells and cells transfected with siRNA or (NS)siRNA for 12h were infected with 10 MOI of KSHV. At the end of 30min PI, KSHV infection was assessed by monitoring ORF50 expression by qRT-PCR.

2. SUPPLEMENTAL FIGURES:

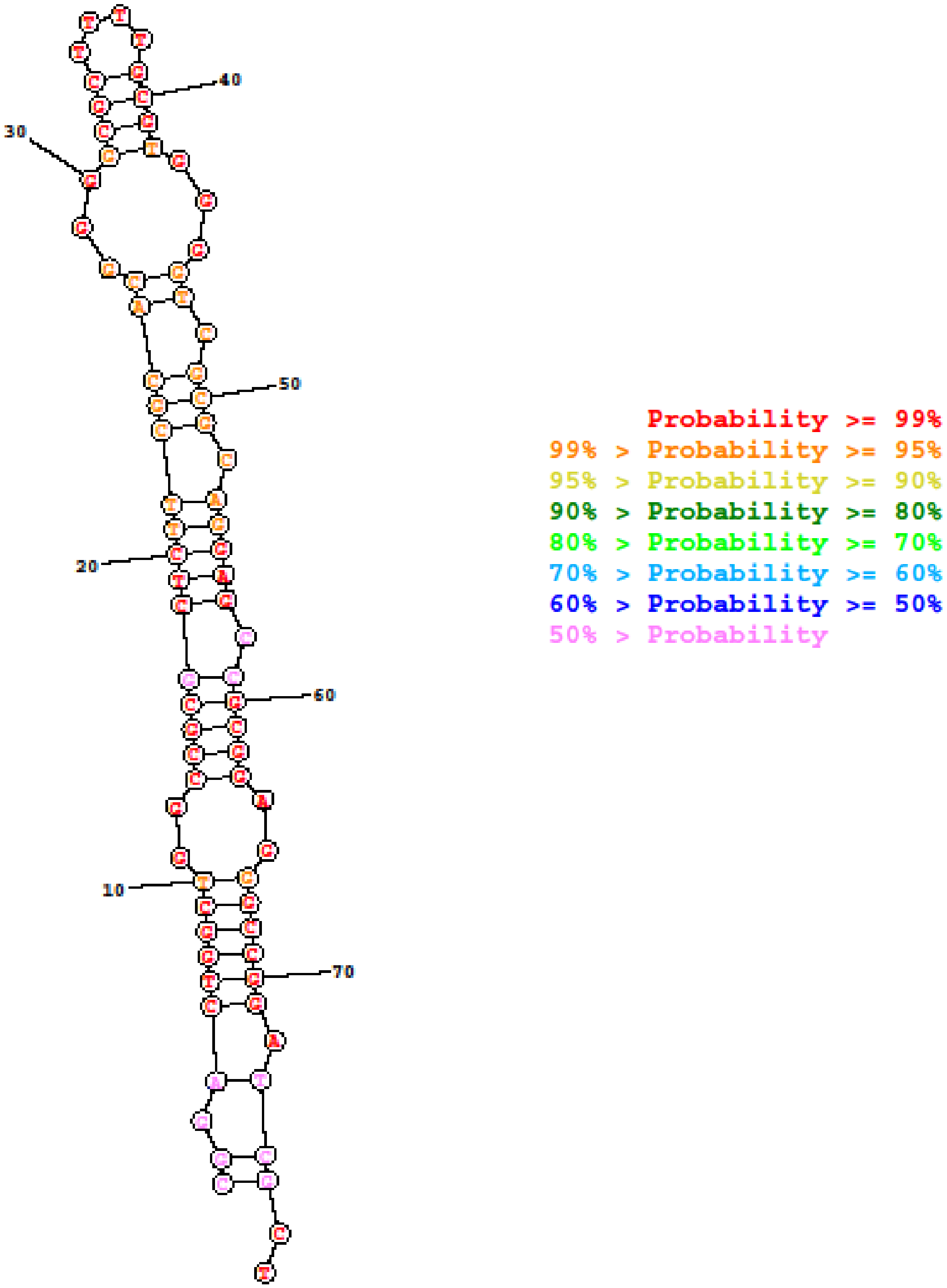

Supplemental Figure 1. Secondary structures of Pre-mir-36. Structure was predicted using the RNAstructure software and base-pairing probability depicted in colors. miR-36 is located at 3p of the duplex[85].

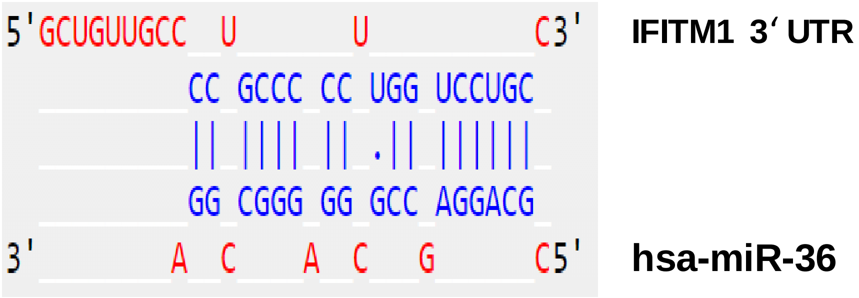

Supplemental Figure 2. RNA hybrid analysis shows the miR-36 binding site located in 3'UTR of IFITM1 mRNA. This is predicted using DIANA and MiRmap algorithms[86,87].

IFITM1 (14kDa)

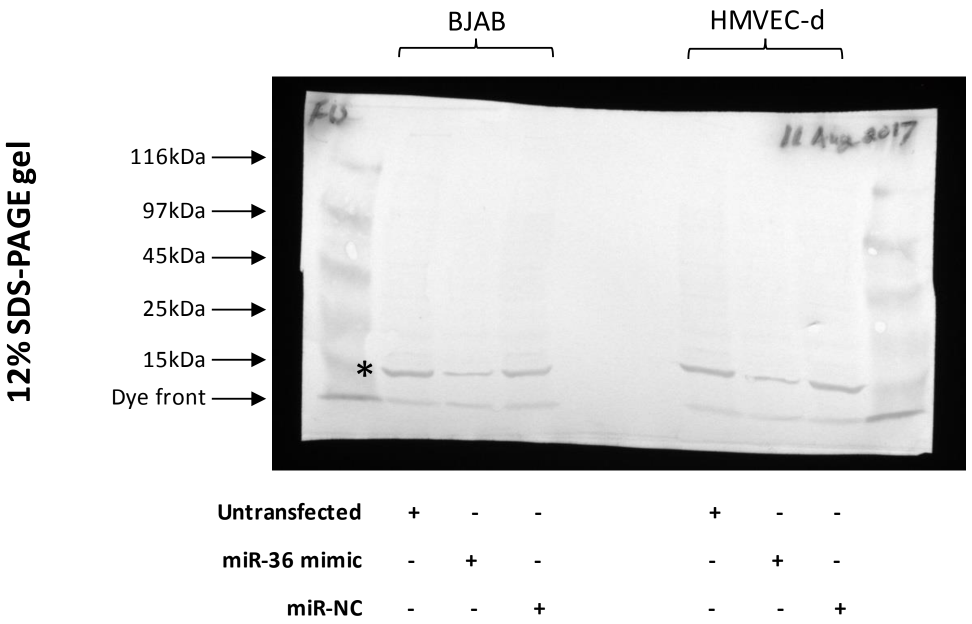

β-actin (42kDa)

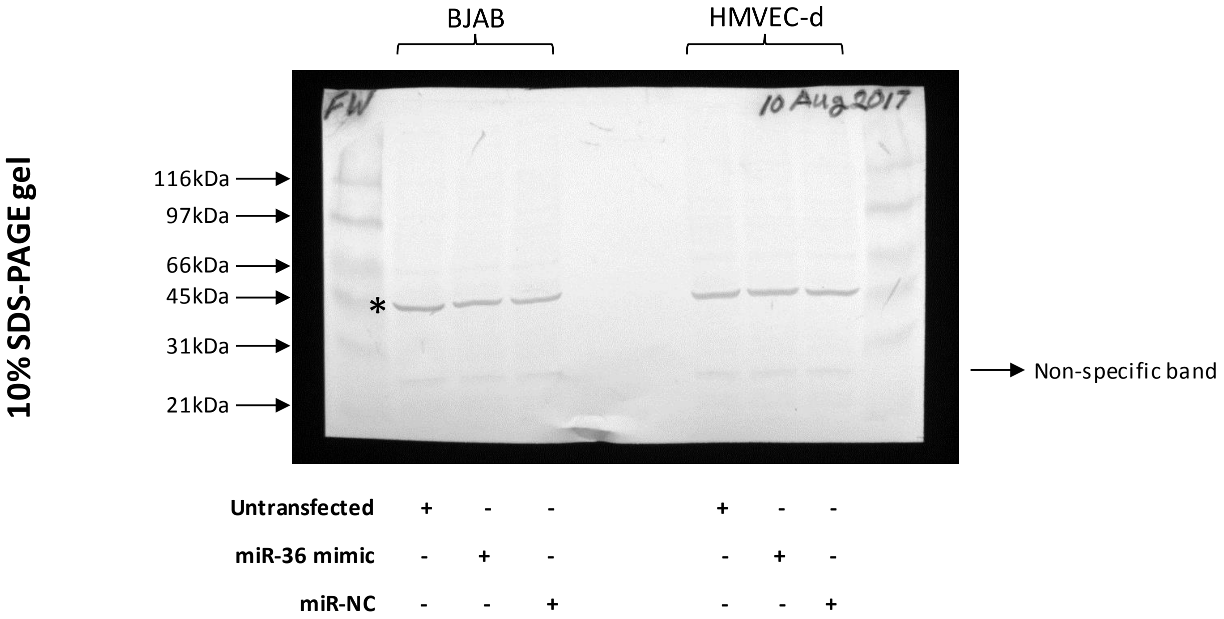

Supplemental Figure 3. Original Western blots of the data presented in Figure 4a.

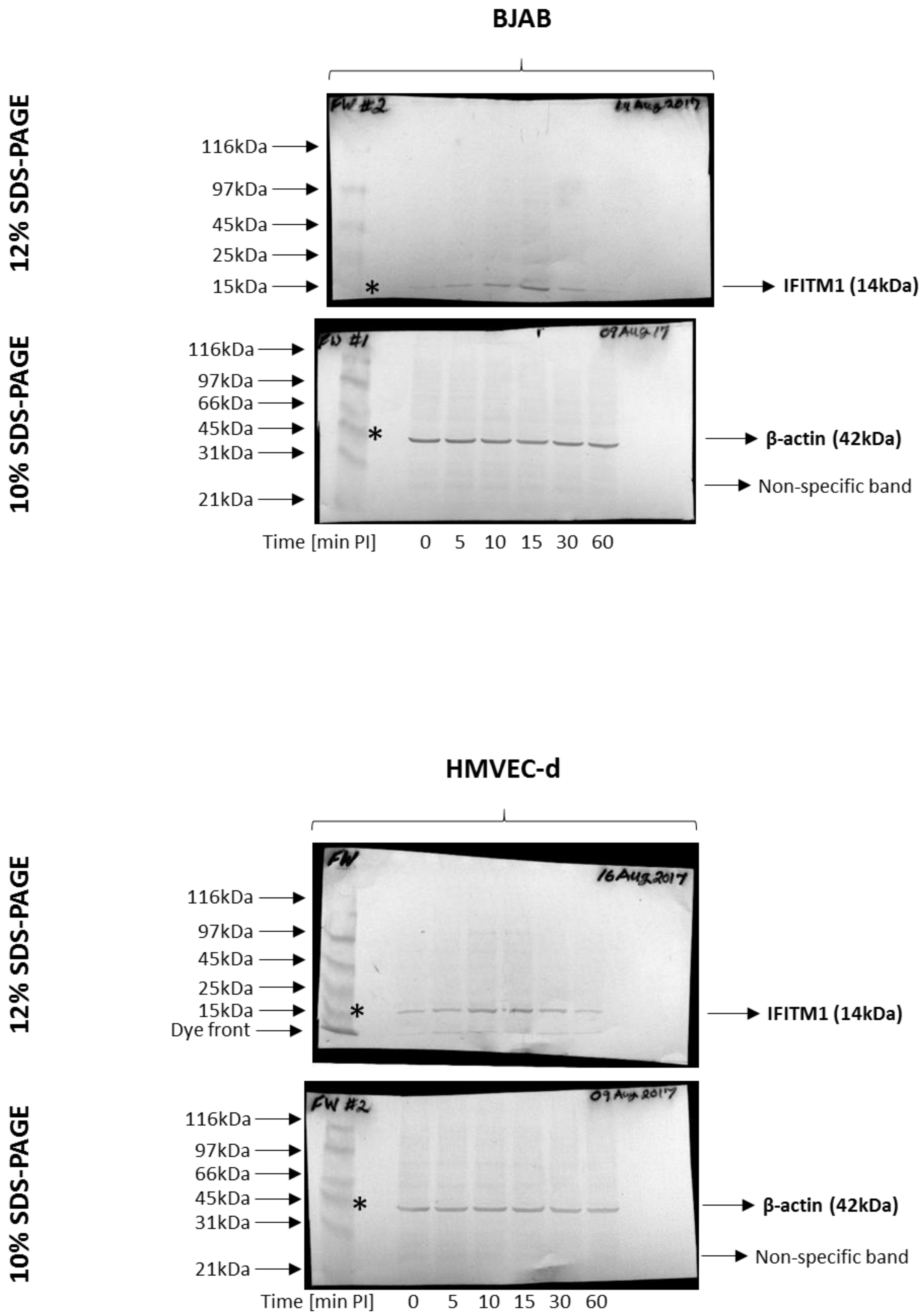

Supplemental Figure 4. Original Western blots of the data presented in Figure 5a.

ORIGINAL BLOTS FOR FIGURE 6A

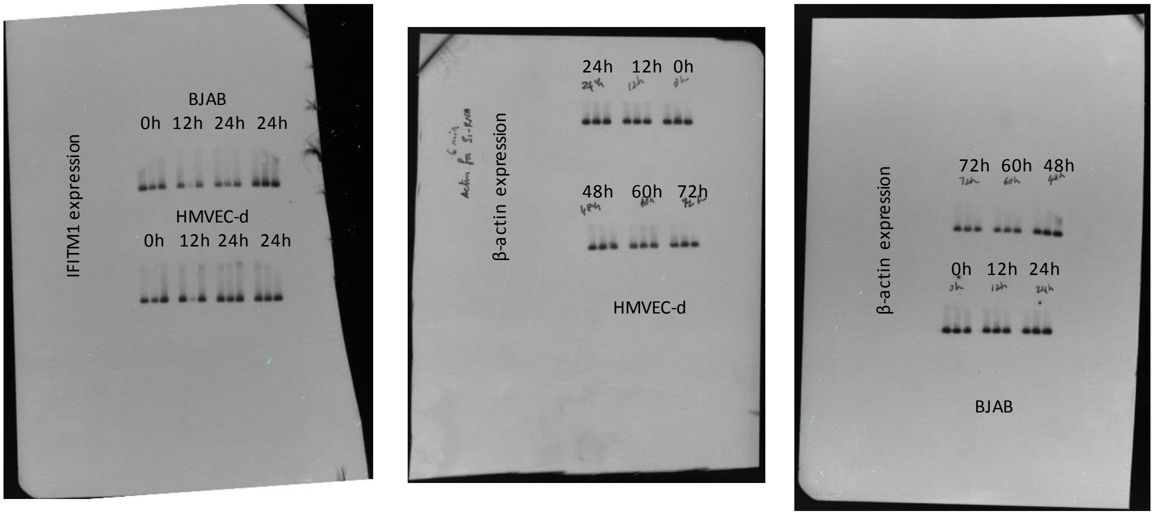

ORIGINAL BLOTS FOR FIGURE 6B

IFITM1

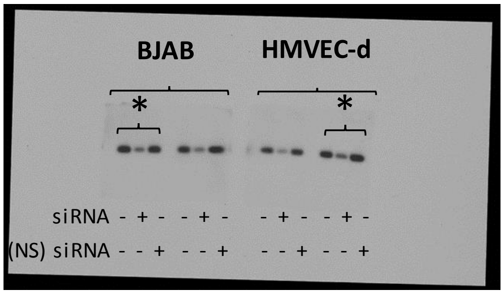

This image has duplicate experiments.
The chosen lanes for the figure is
Indicated by an '*' mark.

β-actin

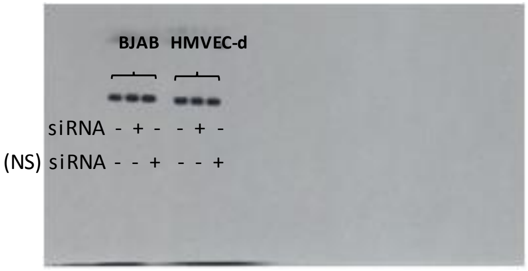

Supplemental Figure 5. Original Northern blots of the data presented in Figure 6a, b.

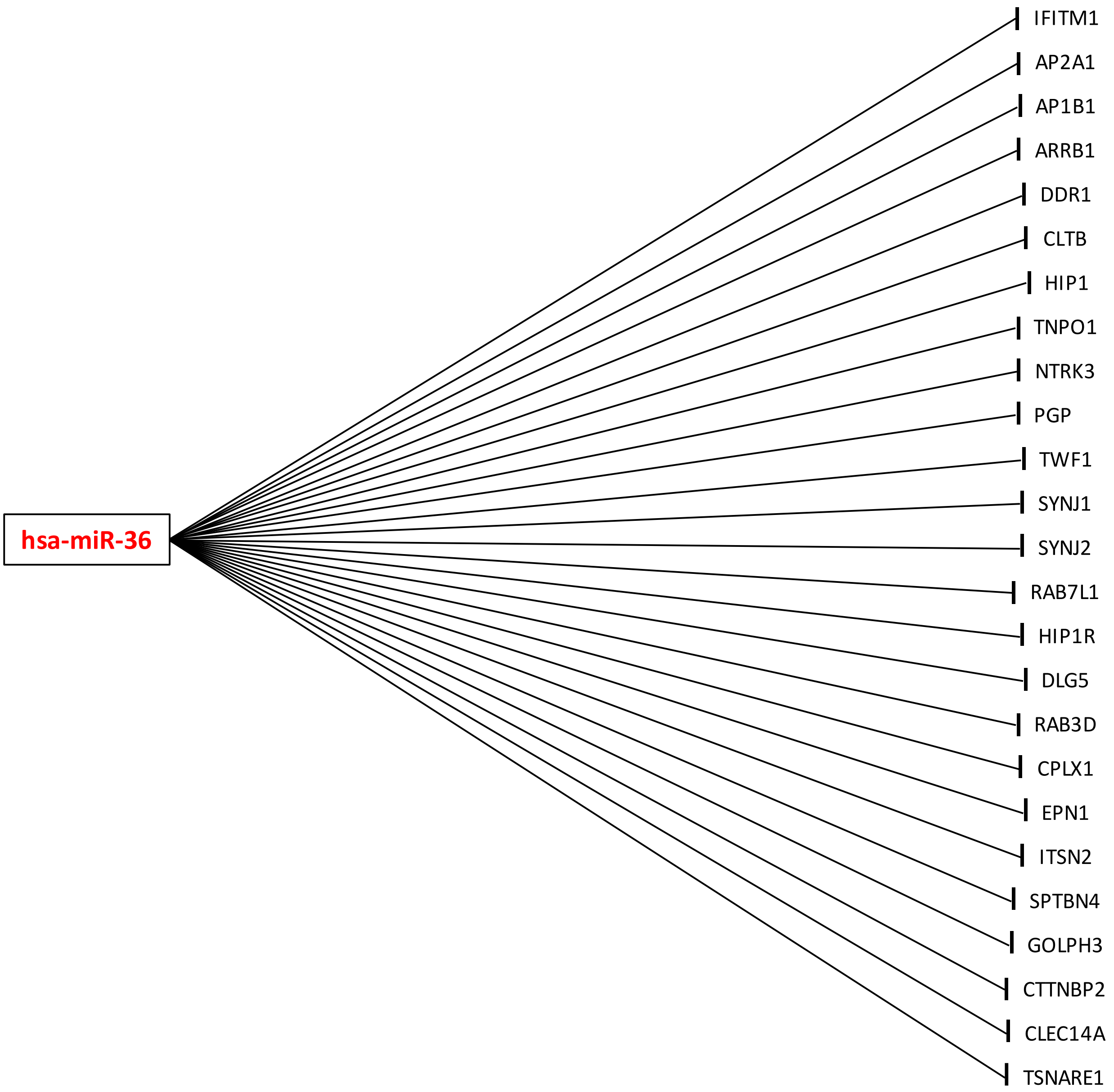

Supplemental Figure 6. Putative target genes for miR-36. miR-36 has been predicted to modulate expression of 25 target genes with confirmed roles during early stages of virus infection. DIANA and MiRamp tools was used for prediction of miR-36 target genes.

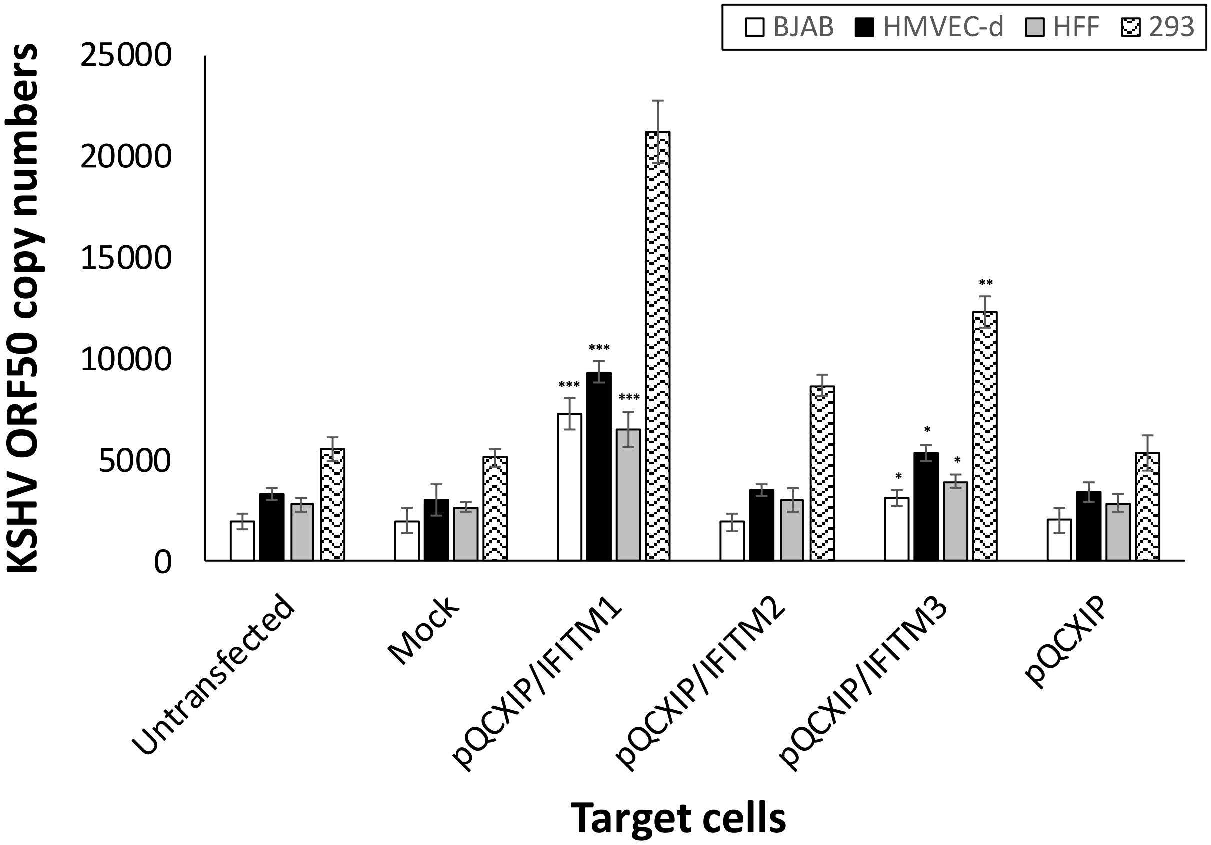

Supplemental Figure 7. IFITM1 and IFITM3 but not IFITM2 enhance KSHV infection of cells. BJAB, HMVEC-d, HFF and 293 cells were either untransfected, mock transfected, transiently transfected with pQCXIP/IFITM1, pQCXIP/IFITM2, pQCXIP/IFITM3, or pQCXIP prior to infecting with 10 MOI of KSHV. Data was plotted to represent the changes in KSHV-*ORF50* RNA copy numbers at 30min PI. Bars represent average ± s.d. of five individual experiments. Student *t* test was performed to compare groups. Two-tailed P value of 0.05 or less was considered statistically significant. *p<0.05; **p,0.01; ***p<0.001; NS-not significant.

(A)

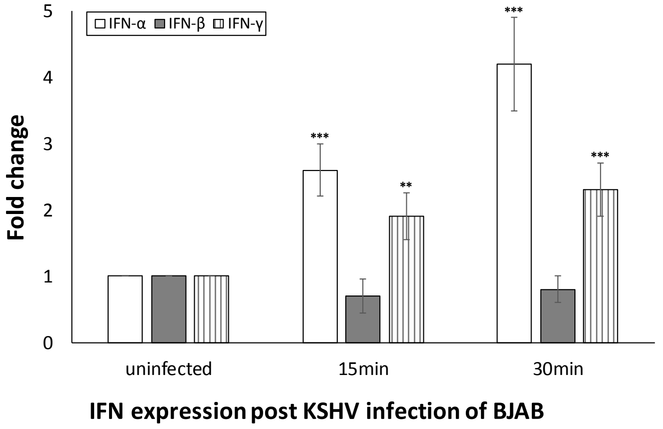

(B)

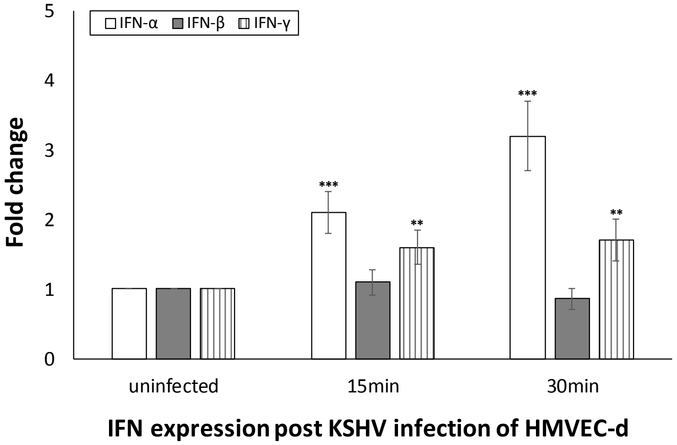

Supplemental Figure 8. KSHV infection of cells induce expression of IFNs in target cells. Expression of IFNs were detected by qRT-PCR at different time points post-infection in BJAB (**A**) and HMVEC-d (**B**) cells infected with 10 MOI of KSHV compared to uninfected cells. The qRT-PCR data was plotted for fold changes in the expression of IFNs in cells during the course of infection when compared to the uninfected cells. Bars represent average ± s.d. of five individual experiments. Student *t* test was performed to compare expression of IFNs in uninfected cells versus 15, and 30min PI. Two-tailed P value of 0.05 or less was considered statistically significant. **p,0.01; ***p<0.001.
